# Assessing parameter identifiability in compartmental dynamic models using a computational approach: application to infectious disease transmission models

**DOI:** 10.1186/s12976-018-0097-6

**Published:** 2019-01-14

**Authors:** Kimberlyn Roosa, Gerardo Chowell

**Affiliations:** 1 Department of Population Health Sciences, School of Public Health, Georgia State University, Atlanta, GA USA; 2 Division of International Epidemiology and Population Studies, Fogarty International Center, National Institute of Health, Bethesda, MD USA

**Keywords:** Compartmental models, Parameter identifiability, Uncertainty quantification, Epidemic models, Structural parameter identifiability, Practical parameter identifiability

## Abstract

**Background:**

Mathematical modeling is now frequently used in outbreak investigations to understand underlying mechanisms of infectious disease dynamics, assess patterns in epidemiological data, and forecast the trajectory of epidemics. However, the successful application of mathematical models to guide public health interventions lies in the ability to reliably estimate model parameters and their corresponding uncertainty. Here, we present and illustrate a simple computational method for assessing parameter identifiability in compartmental epidemic models.

**Methods:**

We describe a parametric bootstrap approach to generate simulated data from dynamical systems to quantify parameter uncertainty and identifiability. We calculate confidence intervals and mean squared error of estimated parameter distributions to assess parameter identifiability. To demonstrate this approach, we begin with a low-complexity SEIR model and work through examples of increasingly more complex compartmental models that correspond with applications to pandemic influenza, Ebola, and Zika.

**Results:**

Overall, parameter identifiability issues are more likely to arise with more complex models (based on number of equations/states and parameters). As the number of parameters being jointly estimated increases, the uncertainty surrounding estimated parameters tends to increase, on average, as well. We found that, in most cases, R_0_ is often robust to parameter identifiability issues affecting individual parameters in the model. Despite large confidence intervals and higher mean squared error of other individual model parameters, R_0_ can still be estimated with precision and accuracy.

**Conclusions:**

Because public health policies can be influenced by results of mathematical modeling studies, it is important to conduct parameter identifiability analyses prior to fitting the models to available data and to report parameter estimates with quantified uncertainty. The method described is helpful in these regards and enhances the essential toolkit for conducting model-based inferences using compartmental dynamic models.

**Electronic supplementary material:**

The online version of this article (10.1186/s12976-018-0097-6) contains supplementary material, which is available to authorized users.

## Background

Mathematical modeling is commonly applied in outbreak investigations for analyzing mechanisms behind infectious disease transmission and explaining patterns in epidemiological data [1, 2]. Models also provide a quantitative framework for assessing intervention and control strategies and generating epidemic forecasts in real time. However, the successful application of mathematical modeling to investigate epidemics depends upon our ability to reliably estimate key transmission and severity parameters, which are critical for guiding public health interventions. In particular, parameter estimates for a given system are subject to two major sources of uncertainty: noise in the data and assumptions built in the model [3]. Ignoring this uncertainty can result in misleading inferences and potentially incorrect public health policy decisions.

Appropriate and flexible approaches for estimating parameters from data, evaluating parameter and model uncertainty, and assessing goodness of fit are gaining increasing attention [4–8]. For instance, model parameters can be estimated by connecting models with observed data through various methods, including least-squares fitting [9], maximum likelihood estimation [10, 11], and approximate Bayesian computation [12, 13]. An important, yet often overlooked step in estimating parameters is examining parameter identifiability – whether a set of parameters can be uniquely estimated from a given model and data set [14]. Lack of identifiability, or non-identifiability, occurs when multiple sets of parameter values yield a very similar model fit to the data. Non-identifiability may be attributed to the model structure (structural identifiability) or due to the lack of information in a given data set (practical identifiability), which could be associated with the number of observations, spatial-temporal resolution (e.g., daily versus weekly data), and observation error. A parameter set is considered structurally identifiable if any set of parameter values can be uniquely mapped to a model output [15]. As such, structural identifiability is the first step in understanding which model parameters can be estimated from data of certain state(s) of the system at a specific spatial-temporal resolution. Structurally identifiable parameters may still be non-identifiable in practice due to a lack of information in available data. The so-called “practical identifiability” considers real-world data issues: amount of noise in the data and sampling frequency (e.g., data collection process) [14].

Several methods have been proposed to examine structural identifiability of a model without the need of experimental data; these include Taylor series methods [15, 16], differential algebra-based methods [17, 18], and other mathematical approaches [15, 19]. These methods tend to work better in the context of simple rather than complex models. Model complexity, in general, is a function of the number of parameters necessary to characterize the states of the system and the spectrum of dynamics that can be recovered from the model. Model complexity affects the ability to reliably parameterize the model given the available data [3], so there is a need for flexible, mathematically-sound approaches to address parameter identifiability in models of varying complexity. Here, we present a general computational method for quantifying parameter uncertainty and assessing parameter identifiability through a parametric bootstrap approach. We demonstrate this approach through examples of compartmental epidemic models with variable complexity, which have been previously employed to study the transmission dynamics and control of various infectious diseases including pandemic influenza, Ebola, and Zika.

## Methods

### Compartmental models

Compartmental models are widely used in epidemiological literature as a population-level modeling approach that subdivides the population into classes according to their epidemiological status [1, 20]. Compartmental dynamic models are specified by a set of ordinary differential equations and parameters that track the temporal progression of the number of individuals in each of the states of the system [3, 21]. Dynamic models follow the general form:

$$ {\dot{x}}_1(t)={f}_1\left({x}_1,{x}_2,\dots, {x}_h,\Theta \right) $$

$$ {\dot{x}}_2(t)={f}_2\left({x}_1,{x}_2,\dots, {x}_h,\Theta \right) $$

⁞

$$ {\dot{x}}_h(t)={f}_h\left({x}_1,{x}_2,\dots, {x}_h,\Theta \right) $$

Where ${\dot{x}}_i$ is the rate of change of the system states (where *i* = 1, 2, …, h) and Θ = (θ_1_, θ_2_, …, θ_m_) is the set of model parameters.

The basic reproductive number (denoted R_0_) is often a parameter of interest in epidemiological studies, as it is a measure of potential for a given infectious disease to spread within a population. Mathematically, it is defined as the average number of secondary infections produced by a single index case in a completely susceptible population [22]. R_0_ represents an epidemic threshold for which values of R_0_ < 1 indicate a lack of disease spread, and values of R_0_ > 1 are consistent with epidemic spread. In the midst of an epidemic, R_0_ estimates provide insight to the intensity of interventions required to achieve control [23]. R_0_ is a composite parameter value, as it depends on multiple model parameters (e.g., transmission rate, infectious period), and while R_0_ is not directly estimated from the model, it can be calculated by relying on the uncertainty of individual parameters.

A simple and commonly utilized compartmental model is the SEIR (susceptible-exposed-infectious-removed) model [1]. We apply our methodology to this low-complexity model and work through increasingly more complex models as we demonstrate the approach for assessing parameter identifiability.

### Model 1: Simple SEIR (pandemic influenza)

**Fig. 1:**
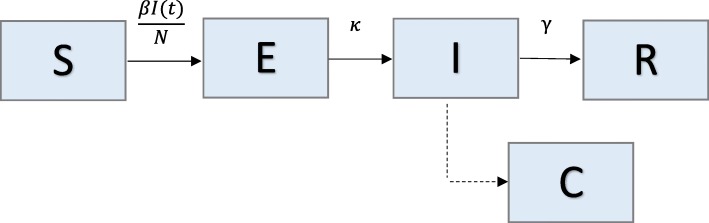
Model 1: Simple SEIR – Population is divided into 4 classes: susceptible (S), exposed (E), infectious (I), and recovered/removed (R). Class C represents the auxiliary variable C (t) and tracks the cumulative number of infectious individuals from the start of the outbreak. This is presented as a dashed line, as it is not a state of the system of equations, but simply a class to track the cumulative incidence cases; meaning, individuals from the population are not moving to class C. Parameter(s) above arrows denote the rate individuals move between classes. Parameter descriptions and values are found in Table 1

We analyze a simple compartmental transmission model that consists of 4 parameters and 4 states (Fig. 1). We apply this model to the context of the 1918 influenza pandemic in San Francisco, California [23]. Individuals in the model are classified as susceptible (S), exposed (E), infectious (I), or recovered (R) [1]. We assume constant population size, so S + E + I + R = N, where N is the total population size. Susceptible individuals progress to the exposed class at rate *βI*(*t*)/*N*, where *β* is the transmission rate, and *I*(*t*)/*N* is the probability of random contact with an infectious individual. Exposed, or latent, individuals move to the infectious class at rate *k*, where 1/*k* is the average latent period. Infectious individuals recover (move to recovered class) at rate *γ*, where 1/*γ* corresponds to the average infectious period.

The transmission process can be modeled using the following system of ordinary differential equations (where the dot denotes time derivative):

$$ \left\{\begin{array}{c}\dot{S}(t)=-\beta S(t)I(t)/N\ \\ {}\dot{E}(t)=\beta S(t)I(t)/N- kE(t)\ \\ {}\dot{I}(t)= kE(t)-\gamma I(t)\ \\ {}\dot{R}(t)=\gamma I(t)\ \\ {}\dot{C}(t)= kE(t)\ \end{array}\right. $$

The auxiliary variable C(t) tracks the cumulative number of infectious individuals from the start of the outbreak. It is not a state of the system of equations, but simply a class to track the cumulative incidence cases; meaning, individuals from the population are not moving to class C. The number of new infections, or the incidence curve, is given by $\dot{C}(t)$.

For this model, there is only one class contributing to new infections (I), so R_0_, or the basic reproductive number, is simply the product of the transmission rate and the average infectious period: R_0_ = $\frac{\beta }{\gamma }$ .

### Model 2: SEIR with asymptomatic and hospitalized/diagnosed and reported

**Fig. 2:**
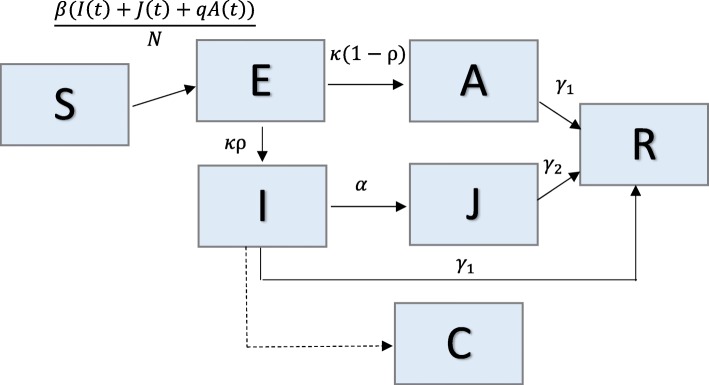
Model 2: SEIR with asymptomatic and hospitalized/diagnosed and reported – Population is divided into 6 classes: susceptible (S), exposed (E), clinically ill and infectious (I), asymptomatic and partially infectious (A), hospitalized/diagnosed and reported (J), and recovered (R). Class C represents the auxiliary variable C(t) and tracks the cumulative number of newly infectious individuals. Parameter(s) above (or to the left of) arrows denote the rate individuals move between classes. Parameter descriptions and values are found in Table 2

We use a simplified version of a complex SEIR model that consists of 8 parameters and 6 system states (Fig. 2). This model was originally developed for studying the transmission dynamics of the 1918 influenza pandemic in Geneva, Switzerland [24]. In the model, individuals are classified as susceptible (S), exposed (E), clinically ill and infectious (I), asymptomatic and partially infectious (A), hospitalized/diagnosed and reported (J), or recovered (R). Hospitalized individuals are assumed to be as infectious as individuals in the I class. Again, constant population size is assumed, so S + E + I + A + J + R = N. Susceptible individuals progress to the exposed class at rate *β*[*I*(*t*) + *J*(*t*) + *qA*(*t*)]/*N*, where *β* is the transmission rate, and q is a reduction factor of transmissibility in the asymptomatic class (0 < q < 1). A proportion, ρ, of exposed/latent individuals (0 < ρ < 1) become clinically infectious at rate *k*, while the rest (1- ρ) become partially infectious and asymptomatic at the same rate *k*. Asymptomatic cases progress to the recovered class at rate *γ*_1_. Clinically ill and infectious individuals are diagnosed at a rate α or recover without being diagnosed at rate *γ*_1_. Diagnosed individuals recover at rate *γ*_2_.

The transmission process can be modeled using the following system of ordinary differential equations:

$$ \left\{\begin{array}{c}\dot{S}(t)=-\beta S(t)\left[I(t)+J(t)+ qA(t)\right]/N\ \\ {}\dot{E}(t)=\beta S(t)\left[I(t)+J(t)+ qA(t)\right]/N- kE(t)\\ {}\dot{A}(t)=k\left(1-\rho \right)E(t)-{\gamma}_1A(t)\\ {}\dot{I}(t)= k\rho E(t)-\left(\alpha +{\gamma}_1\right)I(t)\\ {}\dot{J}(t)=\alpha I(t)-{\gamma}_2J(t)\\ {}\dot{R}(t)={\gamma}_1\left(A(t)+I(t)\right)+{\gamma}_2J(t)\\ {}\dot{C}(t)=\alpha I(t)\end{array}\right. $$

In the above system, C(t) represents the cumulative number of diagnosed/reported cases from the start of the outbreak, and $\dot{C}(t)$ is the incidence curve of diagnosed cases.

For this model, there are three classes contributing to new infections (A, I, J), so the reproductive number is the sum of the contributions from each of these classes: R_0_ = R_0_^A^ + R_0_^I^ + R_0_^J^, where:

R_0_^A^ = (fraction of asymptomatic cases) x (transmission rate) x (relative transmissibility from asymptomatic cases) x (mean time in asymptomatic class)

R_0_^I^ = (fraction of symptomatic cases) x (transmission rate) x (mean time in clinically infectious class)

R_0_^J^ = (fraction of symptomatic cases that are hospitalized) x (transmission rate) x (mean time in hospital) [24]

Here, $$ {R}_0=\beta \Big[\left(1-\rho \right)\left(\frac{q}{\gamma_1}\right)+\rho \left(\frac{1}{\gamma_1+\alpha }+\frac{\alpha }{\left({\gamma}_1+\alpha \right){\gamma}_2}\right) $$].

### Model 3: The Legrand et al. model (Ebola)

**Fig. 3:**
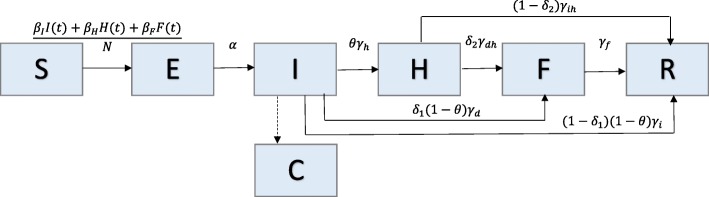
Model 3: The Legrand et al. Model – Population is divided into 6 classes: susceptible (S), exposed (E), infectious in the community (I), infectious in the hospital (H), infectious after death at funeral (F), or recovered/removed (R). Class C represents the auxiliary variable C(t) and tracks the cumulative number of newly infectious individuals. Parameter(s) above arrows denote the rate that individuals move between classes. Parameter descriptions and values are found in Table 3

We analyze an Ebola transmission model [25] comprised of 15 parameters and 6 states (Fig. 3). This model subdivides the infectious population into three stages to account for transmission in three settings: community, hospital, and unsafe burial ceremonies. Individuals are classified as susceptible (S), exposed (E), infectious in the community (I), infectious in the hospital (H), infectious after death at funeral (F), or recovered/removed (R). Constant population size is assumed, so S + E + I + H + F + R = N. Susceptible individuals progress to the exposed class at rate (*β*_*I*_*I*(*t*) + *β*_*H*_*H*(*t*) + *β*_*F*_*F*(*t*))/*N* where β_I_, β_H_, and β_F_ represent the transmission rates in the community, hospital, and at funerals, respectively. Exposed individuals become infectious at rate α. A proportion, 0 < θ < 1, of infectious individuals are hospitalized at rate γ_h_. Of the proportion of infectious individuals that are not hospitalized (1-θ), a proportion, 0 < δ_1_ < 1, move to the funeral class at rate γ_d_, and the rest (1- δ_1_) move to the recovered/removed class at rate γ_i_. A proportion, 0 < δ_2_ < 1, of hospitalized individuals progress to funeral class at rate ${\upgamma}_{dh}=\frac{1}{\frac{1}{\upgamma_d}-\frac{1}{\upgamma_h}}$. The remaining proportion (1- δ_2_) are recovered/removed at rate ${\upgamma}_{ih}=\frac{1}{\frac{1}{\upgamma_i}-\frac{1}{\upgamma_h}}$. δ_1_ and δ_2_ are calculated such that δ represents the case fatality ratio (Table 3). Individuals in the funeral class are removed at rate γ_f_.

The transmission process is modeled by the following set of ordinary differential equations:

$$ \left\{\begin{array}{c}\dot{S}(t)=-S(t)\left[{\upbeta}_II(t)+{\beta}_HH(t)+{\beta}_FF(t)\right]/N\ \\ {}\dot{E}(t)=S(t)\left[{\beta}_II(t)+{\beta}_HH(t)+{\beta}_FF(t)\right]/N-\alpha E(t)\\ {}\dot{I}(t)=\alpha E(t)-\left[\uptheta {\gamma}_h+{\delta}_1\left(1-\uptheta \right){\gamma}_d+\left(1-{\delta}_1\right)\left(1-\uptheta \right){\gamma}_i\right]I(t)\\ {}\dot{H}(t)=\uptheta {\gamma}_hI(t)-\left[\left(1-{\delta}_2\right){\gamma}_{ih}+{\delta}_2{\gamma}_{dh}\right]H(t)\\ {}\dot{F}(t)={\delta}_1\left(1-\uptheta \right){\gamma}_dI(t)+{\delta}_2{\gamma}_{dh}H(t)-{\gamma}_fF(t)\\ {}\dot{R}(t)=\left(1-{\delta}_1\right)\left(1-\uptheta \right){\gamma}_iI(t)+\left(1-{\delta}_2\right){\gamma}_{ih}H(t)+{\gamma}_fF(t)\\ {}\dot{C}(t)=\alpha E(t)\end{array}\right. $$

Here, C(t) represents the cumulative number of all infectious individuals, and $\dot{C}(t)$ is the incidence curve for infectious cases.

The basic reproductive number is the sum of the contributions from each of the infectious classes (I, H, F): R_0_ = R_0_^I^ + R_0_^H^ + R_0_^F^, where:

R_0_^I^ = (transmission rate in the community) x (mean time in infectious class)

R_0_^H^ = (fraction of hospitalized cases) x (transmission rate in the hospital) x (mean time in hospital class)

R_0_^F^ = (fraction of cases that have traditional burial ceremonies) x (transmission rate at funerals) x (mean time in funeral class)

Here, $$ {R}_0=\frac{\beta_I}{\Delta  }+\frac{\frac{\gamma_h\theta }{\gamma_{dh}{\delta}_2+{\gamma}_{ih}\left(1-{\delta}_2\right)}{\beta}_H}{\Delta  }+\frac{\gamma_d{\delta}_1\left(1-\theta \right){\beta}_F}{\gamma_f\Delta  }+\frac{\gamma_{dh}{\gamma}_h{\delta}_2\theta {\beta}_F}{\gamma_f\left({\gamma}_{ih}\left(1-{\delta}_2\right)+{\gamma}_{dh}{\delta}_2\right)\Delta  }, $$

where *∆* = *γ*_*h*_*θ* + *γ*_*d*_(1 − *θ*)*δ*_1_ + *γ*_*i*_(1 − *θ*)(1 − *δ*_1_) [25].

### Model 4: Zika model with human and mosquito populations

**Fig. 4:**
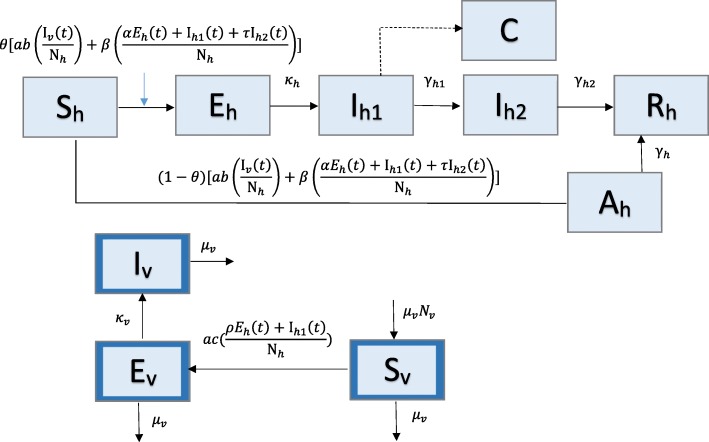
Model 4: Zika Model with human and mosquito populations – The human population (subscript h) is divided into 5 classes: susceptible (S_h_), asymptomatically infected (A_h_), exposed (E_h_), symptomatically infectious (I_h1_), convalescent (I_h2_), or recovered (R_h_). Class C represents the auxiliary variable C(t) and tracks the cumulative number of newly infectious individuals. The mosquito, or vector, population (subscript v; outlined in dark blue) is divided into 3 classes: susceptible (S_v_), exposed (E_v_), and infectious (I_v_) classes. Parameter(s) above arrows denote the rate individuals/vectors move between classes. Parameter descriptions and values are found in Table 4

The last example is a compartmental model of Zika transmission dynamics that includes 16 parameters and 9 states and incorporates transmission between two populations – humans and vectors (Fig. 4). This model was designed to investigate the impact of both mosquito-borne and sexually transmitted (human-to-human) routes of infection for cases of Zika virus [26]. In the human population, individuals are classified as susceptible (S_h_), asymptomatically infected (A_h_), exposed (E_h_), symptomatically infectious (I_h1_), convalescent (I_h2_), or recovered (R_h_). The mosquito, or vector, population is broken into susceptible (S_v_), exposed (E_v_), and infectious (I_v_) classes. Note that the subscript ‘h’ is used for humans and ‘v’ is used for vectors. Constant population size is assumed in both populations, so S_h_ + A_h_ + E_h_ + I_h1_ + I_h2_ + R_h_ = N_h_ and S_v_ + E_v_ + I_v_ = N_v_.

A proportion 0 < θ < 1 of susceptible humans move to the exposed class at rate *ab*(I_*v*_(*t*)/N_*h*_) + *β*[(*αE*_*h*_(*t*) + I_*h*1_(*t*) + *τ*I_*h*2_(*t*))/N_*h*_)] where a is the mosquito biting rate, b is the transmission probability from an infectious mosquito to a susceptible human, β is the transmission rate between humans, α is the relative (human-to-human) transmissibility from exposed humans to susceptible, and τ is the relative transmissibility from convalescent humans compared to susceptible. Exposed individuals progress to symptomatically infectious at rate κ_h_ and then progress to the convalescent stage at rate γ_h1_. Convalescent individuals recover at rate γ_h2_. The remaining proportion of susceptible individuals (1 - θ) become asymptomatically infected at the same rate, *ab*(I_*v*_(*t*)/N_*h*_) + *β*[(*αE*_*h*_(*t*) + I_*h*1_(*t*) + *τ*I_*h*2_(*t*))/N_*h*_]. Asymptomatic humans recover at rate γ_h_ and do not contribute to new infections in this model.

Susceptible mosquitos move to the exposed class at rate *ac*[(*ρE*_*h*_(*t*) + I_*h*1_(*t*))/N_*h*_], where c is the transmission probability from a symptomatically infectious human to a susceptible mosquito, and ρ is the relative human-to-mosquito transmission probability from exposed humans to symptomatically infected. Exposed mosquitos become infectious at rate κ_v_. Mosquitos also leave the population at rate μ_v_, where 1/μ_v_ is the mosquito lifespan.

The transmission process, including both populations, is represented by the set of differential equations below:

$$ \left\{\begin{array}{c}{\dot{S}}_h(t)=- ab\left({\mathrm{I}}_v(t)/{\mathrm{N}}_h\right){\mathrm{S}}_h(t)-\beta \left[\left(\alpha {E}_h(t)+{\mathrm{I}}_{h1}(t)+\tau {\mathrm{I}}_{h2}(t)\right)/{\mathrm{N}}_h\right]{\mathrm{S}}_h(t)\\ {}{\dot{E}}_h(t)=\theta \left[ ab\left({\mathrm{I}}_v(t)/{\mathrm{N}}_h\right){\mathrm{S}}_h(t)+\beta \left[\left(\alpha {E}_h(t)+{\mathrm{I}}_{h1}(t)+\tau {\mathrm{I}}_{h2}(t)\right)/{\mathrm{N}}_h\right]{\mathrm{S}}_h(t)\right]-{\kappa}_h{E}_h(t)\\ {}{\dot{I}}_{h1}(t)={\kappa}_h{E}_h(t)-{\gamma}_{h1}{\mathrm{I}}_{h1}(t)\\ {}{\dot{I}}_{h2}(t)={\gamma}_{h1}{\mathrm{I}}_{h1}(t)-{\gamma}_{h2}{\mathrm{I}}_{h2}(t)\\ {}{\dot{A}}_h(t)=\left(1-\theta \right)\left[ ab\right({\mathrm{I}}_v(t)/{\mathrm{N}}_h\left]{\mathrm{S}}_h(t)+\beta \left[\alpha {E}_h(t)+{\mathrm{I}}_{h1}(t)+\tau {\mathrm{I}}_{h2}(t)\Big)/{\mathrm{N}}_h\right]{\mathrm{S}}_h(t)\right]-{\gamma}_h{A}_h(t)\\ {}{\dot{R}}_h(t)={\gamma}_{h2}{\mathrm{I}}_{h2}(t)+{\gamma}_h{A}_h(t)\\ {}{\dot{S}}_v(t)={\mu}_v{N}_v- ac\left[\left(\rho {E}_h(t)+{\mathrm{I}}_{h1}(t)\right)/{\mathrm{N}}_h\right]\ast {\mathrm{S}}_v(t)-{\mu}_v{\mathrm{S}}_v(t)\\ {}{\dot{E}}_v(t)= ac\left[\left(\rho {E}_h(t)+{\mathrm{I}}_{h1}(t)\right)/{\mathrm{N}}_h\right]\ast {\mathrm{S}}_v(t)-\left({\kappa}_v+{\mu}_v\right){\mathrm{E}}_v(t)\\ {}{\dot{I}}_v(t)={\kappa}_v{\mathrm{E}}_v(t)-{\mu}_v{\mathrm{I}}_v(t)\\ {}\dot{C}(t)={\kappa}_h{E}_h(t)\end{array}\right. $$

C(t) represents the cumulative number of symptomatically infectious human cases, and $\dot{C}(t)$ contains the incidence curve for symptomatic human cases.

For this example, we have two transmission processes to consider when calculating R_0_: sexual transmission (R_hh_) and mosquito-borne (R_hv_). The human population has three classes contributing to new infections: exposed, symptomatically infectious, and convalescent, so:

$$ {R}_{hh}=\frac{\alpha \theta \beta}{\kappa_h}+\frac{\theta \beta}{\gamma_{h1}}+\frac{\tau \theta \beta}{\gamma_{h2}} $$

The mosquito population only has one infectious class (I_v_); the reproductive number is given by:

$$ {R}_{hv}=\sqrt{\left[\frac{a^2 b\rho cm\theta}{\kappa_h{\mu}_v}+\frac{a^2 b cm\theta}{\gamma_{h1}{\mu}_v}\right]\ast \frac{\kappa_v}{\kappa_v+{\mu}_v}}. $$

The overall basic reproductive number, considering both transmission routes, is given by the following eq. [26]:

$$ {R}_0=\frac{R_{hh}+\sqrt{R_{hh}^2+4{R}_{hv}^2}}{2} $$

### Simulated data

For each model we simulate 200 epidemic datasets (directly from the corresponding set of ordinary differential equations) with Poisson error structure using the daily time series data of case incidence, or total number of new cases daily. Parameters for each model are set at values based on their corresponding application: the 1918 influenza pandemic in San Francisco (Model 1) [23], 1918 pandemic influenza in Geneva (Model 2) [24], 1995 Ebola in Congo (Model 3) [25], and 2016 Zika in the Americas (Model 4) [26]. As explained below, the simulated data are generated using a bootstrap approach, and we then use these data to study parameter identifiability within a realistic parameter space for each model. Parameter descriptions and their corresponding values for each model are given in Tables 1, 2, 3 and 4.

**Table 1:** Parameter descriptions and values for Model 1

Parameters	Description	Value
N	Population size	500,000
β	Transmission rate (per day)	0.56
1/κ	Mean latent period (days)	1.9
1/γ	Mean infectious period (days)	4.1
R_0_	Basic reproductive number	2.3

Parameter values are consistent with pandemic influenza in San Francisco, 1918 [23]

**Table 2:** Parameter descriptions and values for Model 2

Parameters	Description	Value
N	Population size	500,000
β	Transmission rate (per day)	0.8
1/κ	Latent period (days)	1.9
γ_1_	Recovery rate for asymptomatic individuals (1/days)	1/4.1
γ_2_	Recovery rate for infectious individuals recovering without hospitalization (1/days)	1/2.3
α	Rate of diagnosis for hospitalized individuals (days)	0.555
ρ	Proportion of latent individuals progressing to infectious class (vs. asymptomatic class)	0.6
q	Reduction factor in transmissibility for asymptomatic cases	0.4
R_0_	Basic reproductive number	1.89

Parameter values are consistent with pandemic influenza in Geneva, 1918 [24]

**Table 3:** Parameter descriptions and values for Model 3

Parameters	Description	Value
N	Population size	200,000
β_I_	Transmission rate in the community (per day)	0.084
β_H_	Transmission rate in the hospital (per day)	0.1134
β_F_	Transmission rate at traditional funerals (per day)	1.093
1/α	Incubation period (days)	7
θ	Proportion of cases hospitalized	0.80
1/γ_h_	Time from symptom onset to hospitalization (days)	5
1/γ_d_	Time from symptom onset to death (days)	9.6
1/γ_i_	Time from symptom onset to the end of infectiousness for survivors (days)	10
δ	Case fatality ratio	0.81
δ_1_	${\updelta}_1=\frac{\updelta {\gamma}_i}{\updelta {\gamma}_i+\left(1-\updelta \right){\gamma}_d}$	0.80
δ_2_	${\updelta}_2=\frac{\updelta {\gamma}_{ih}}{\updelta {\gamma}_{ih}+\left(1-\updelta \right){\gamma}_{dh}}$	0.80
1/γ_ih_	Infectious period for survivors (days)	5
1/γ_dh_	Time from hospitalization to death (days)	4.6
1/γ_f_	Time from death to funeral (days)	2
R_0_	Basic reproductive number	2.685

Parameter values are consistent with the 1995 Ebola outbreak in the Democratic Republic of Congo [25]

**Table 4:** Parameter descriptions and values for Model 4

Parameters	Description	Value
N_h_	Population size (humans)	200,000
N_v_	Population size (mosquitos)	1,000,000
a	Mosquito biting rate (number of bites per mosquito per day)	0.5
b	Probability of infection from an infectious mosquito to a susceptible human (per bite)	0.4
β	Transmission rate from symptomatically infected humans to susceptible humans (per day)	0.05
α	Relative human-to-human transmissibility of exposed humans to symptomatic humans	0.6
τ	Relative human-to-human transmissibility of convalescent to symptomatic humans	0.3
ϴ	Proportion of symptomatic infections	0.18
1/κ_h_	Intrinsic incubation period in humans (days)	5
1/γ_h1_	Duration of acute phase (days)	5
1/γ_h2_	Duration of convalescent phase (days)	20
1/γ_h_	Duration of asymptomatic infection (days)	
1/μ_v_	Mosquito lifespan (days)	14
c	Transmission probability from a symptomatically infected human to a susceptible mosquito per bite	0.5
ρ	Relative human-to-mosquito transmission probability of exposed humans to symptomatically infected humans	0.1
1/κ_v_	Extrinsic incubation period in mosquitos (days)	10
R_0_	Basic reproductive number	1.486

Parameter values are consistent with the 2016 Zika outbreak in Brazil, Colombia, and El Salvador [26]

### Parameter estimation

To estimate parameter values, we fit the model to each simulated dataset using nonlinear least squares estimation. The *lsqcurvefit* function in Matlab (Mathworks, Inc.) is used to find the least squares best fit to the data. This process searches for the set of parameters $\widehat{\varTheta}$= ($\widehat{\theta}$_1_, $\widehat{\theta}$_2_,…, $\widehat{\theta}$_m_) that minimizes the sum of squared differences between the simulated data and the model solution [3]. The model solution $f\left({t}_i,\widehat{\varTheta}\right)$ represents the best fit to the time series data.

For this method, the initial parameter predictions affect the solution for the model as local minima occur. While we know the true parameter values (used to generate the data), this is unrealistic for a real-world modeling scenario. We vary the initial guesses of the parameter values to vary according to a uniform distribution in the range of +/− 0.1 around the true value. Another approach would consist of repeating the least squares fitting procedure several times with different initial parameter guesses and selecting the best model fit.

For each model, the sets of parameters are denoted by Θ_i_, where i represents the number of parameters being jointly estimated. We begin with estimating one model parameter, while fixing the rest, and then increase the number of parameters jointly estimated by one until all parameters of interest are included. Population size, N, is always fixed to the true value. Also, while R_0_ is not being directly estimated from the model, it is a composite parameter that can be calculated using individual parameter estimates.

For each model described above, we explore parameter identifiability for the following sets of parameters. Here, the symbol ^ is used to indicate an estimated parameter, while the absence of this symbol indicates that the parameter is set to its true value from the simulated data.

*(i) Model 1: Simple SEIR*

$$ {\displaystyle \begin{array}{cc}{\Theta}_{\mathrm{i}}:& {\Theta}_1=\left\{\ \widehat{\beta},\kappa, \gamma\ \right\}\\ {}& {\Theta}_2=\left\{\ \widehat{\beta},\kappa, \widehat{\gamma}\ \right\}\\ {}& {\Theta}_3=\left\{\ \widehat{\beta},\widehat{\kappa},\widehat{\gamma}\ \right\}\end{array}} $$

*(ii) Model 2: SEIR with asymptomatic and hospitalized/diagnosed and reported*

$$ {\displaystyle \begin{array}{cc}{\Theta}_{\mathrm{i}}:& {\Theta}_1=\left\{\ \widehat{\beta},\kappa, {\gamma}_1,{\gamma}_2,\alpha, \rho, q\ \right\}\\ {}& {\Theta}_2=\left\{\ \widehat{\beta},\kappa, \widehat{\gamma_1},{\gamma}_2,\alpha, \rho, q\ \right\}\\ {}& {\Theta}_3=\left\{\ \widehat{\beta},\kappa, \widehat{\gamma_1},{\gamma}_2,\widehat{\alpha},\rho, q\ \right\}\\ {}& {\Theta}_4=\left\{\ \widehat{\beta},\kappa, \widehat{\gamma_1},{\gamma}_2,\widehat{\alpha},\widehat{\rho},q\ \right\}\\ {}& {\Theta}_5=\left\{\ \widehat{\beta},\kappa, \widehat{\gamma_1},{\gamma}_2,\widehat{\alpha},\widehat{\rho},\widehat{q}\ \right\}\end{array}} $$

*(iii) Model 3: The Legrand Model (Ebola)*

$$ {\displaystyle \begin{array}{cc}{\Theta}_{\mathrm{i}}:& {\Theta}_1=\left\{\ {\widehat{\beta}}_I,{\beta}_H,{\beta}_F,\alpha, \theta, {\gamma}_h,{\gamma}_d,{\gamma}_i,\delta, {\gamma}_{ih},{\gamma}_{dh},{\gamma}_f\ \right\}\\ {}& {\Theta}_2=\left\{\ {\widehat{\beta}}_I,{\widehat{\beta}}_H,{\beta}_F,\alpha, \theta, {\gamma}_h,{\gamma}_d,{\gamma}_i,\delta, {\gamma}_{ih},{\gamma}_{dh},{\gamma}_f\ \right\}\\ {}& {\Theta}_3=\left\{\ {\widehat{\beta}}_I,{\widehat{\beta}}_H,{\widehat{\beta}}_F,\alpha, \theta, {\gamma}_h,{\gamma}_d,{\gamma}_i,\delta, {\gamma}_{ih},{\gamma}_{dh},{\gamma}_f\ \right\}\\ {}& {\Theta}_4=\left\{\ {\widehat{\beta}}_I,{\widehat{\beta}}_H,{\widehat{\beta}}_F,\alpha, \theta, {\widehat{\gamma}}_h,{\gamma}_d,{\gamma}_i,\delta, {\gamma}_{ih},{\gamma}_{dh},{\gamma}_f\ \right\}\\ {}& {\Theta}_5=\left\{\ {\widehat{\beta}}_I,{\widehat{\beta}}_H,{\widehat{\beta}}_F,\alpha, \theta, {\widehat{\gamma}}_h,{\widehat{\gamma}}_d,{\gamma}_i,\delta, {\gamma}_{ih},{\gamma}_{dh},{\gamma}_f\ \right\}\\ {}& {\Theta}_6=\left\{\ {\widehat{\beta}}_I,{\widehat{\beta}}_H,{\widehat{\beta}}_F,\alpha, \theta, {\widehat{\gamma}}_h,{\widehat{\gamma}}_d,{\widehat{\gamma}}_i,\delta, {\gamma}_{ih},{\gamma}_{dh},{\gamma}_f\ \right\}\\ {}& {\Theta}_7=\left\{\ {\widehat{\beta}}_I,{\widehat{\beta}}_H,{\widehat{\beta}}_F,\alpha, \theta, {\widehat{\gamma}}_h,{\widehat{\gamma}}_d,{\widehat{\gamma}}_i,\delta, {\gamma}_{ih},{\gamma}_{dh},{\widehat{\gamma}}_f\ \right\}\end{array}} $$

*(iv) Model 4: Zika model with human and mosquito populations*

$$ {\displaystyle \begin{array}{cc}{\Theta}_{\mathrm{i}}:& {\Theta}_1=\left\{\ a,b,\widehat{\beta},\alpha, \tau, \theta, {\kappa}_h,{\gamma}_{h1},{\gamma}_{h2},{\gamma}_h,{\mu}_v,c,\rho, {\kappa}_v\ \right\}\\ {}& {\Theta}_2=\left\{\ a,b,\widehat{\beta},\alpha, \tau, \theta, {\kappa}_h,{\widehat{\gamma}}_{h1},{\gamma}_{h2},{\gamma}_h,{\mu}_v,c,\rho, {\kappa}_v\ \right\}\\ {}& {\Theta}_3=\left\{\ a,b,\widehat{\beta},\alpha, \tau, \theta, {\kappa}_h,{\widehat{\gamma}}_{h1},{\widehat{\gamma}}_{h2},{\gamma}_h,{\mu}_v,c,\rho, {\kappa}_v\ \right\}\\ {}& {\Theta}_4=\left\{\ a,b,\widehat{\beta},\alpha, \tau, \theta, {\kappa}_h,{\widehat{\gamma}}_{h1},{\widehat{\gamma}}_{h2},{\widehat{\gamma}}_h,{\mu}_v,c,\rho, {\kappa}_v\ \right\}\\ {}& {\Theta}_5=\left\{\ a,b,\widehat{\beta},\widehat{\alpha},\tau, \theta, {\kappa}_h,{\widehat{\gamma}}_{h1},{\widehat{\gamma}}_{h2},{\widehat{\gamma}}_h,{\mu}_v,c,\rho, {\kappa}_v\ \right\}\\ {}& {\Theta}_6=\left\{\ a,b,\widehat{\beta},\widehat{\alpha},\widehat{\tau},\theta, {\kappa}_h,{\widehat{\gamma}}_{h1},{\widehat{\gamma}}_{h2},{\widehat{\gamma}}_h,{\mu}_v,c,\rho, {\kappa}_v\ \right\}\end{array}} $$

### Bootstrapping method

We use the parametric bootstrap approach [3, 27, 28] for simulating the error structure around the deterministic model solution in order to evaluate parameter identifiability. This computational approach involves repeatedly sampling observations from the best-fit model solution. Here we use a Poisson error structure, which is the most popular distribution for modeling count data [3]. The step-by-step approach to quantify parameter uncertainty is as follows:

1. Obtain the deterministic model solution (total daily incidence series) using nonlinear least-squares estimation (*Section 2.3*).
2. Generate S replicate datasets, assuming Poisson error structure:
  Using the deterministic model solution $f\left({t}_i,\widehat{\varTheta}\right)$, generate S (for our examples, S = 200) replicate simulated datasets ${f}_S^{\ast}\left({t}_i,\widehat{\varTheta}\right)$. To incorporate Poisson error structure, we use the incidence curve, $\dot{C}(t)$, as follows. For each time point t, we generate a new incidence value using a Poisson random variable with mean=$\dot{C}(t)$. This new set of data represents an incidence curve for the system, assuming the time series follows a Poisson distribution centered on the mean at time points t_i_.
3. Re-estimate model parameters: For each simulated dataset, derive the best-fit estimates for the parameter set using least-squares fitting (*Section 2.3*). This results in S estimated parameter sets: $\widehat{\varTheta}$_i_ where *i* = 1, 2, …, S.
4. Characterize empirical distributions and construct confidence intervals: Using the set of S parameter estimates, we can characterize the empirical distribution and construct confidence intervals for each estimated parameter. Also, for each set of estimated parameters, R_0_ is calculated to obtain a distribution of R_0_ values as well.

### Parameter identifiability

When a model parameter is identifiable from available data, its confidence interval lies in a finite range of values [29, 30]. Using the bootstrapping method outlined in *Section 2.4*, we obtain 95% confidence intervals from the distributions of each estimated parameter. A small confidence interval with a finite range of values indicates that the parameter can be precisely identified, while a wider range could be indicative of lack of identifiability. To assess the level of bias of the estimates, we calculate the mean squared error (MSE) for each parameter. MSE is calculated as: $MSE=\frac{1}{S}\sum \limits_{i=1}^S{\left(\uptheta -\widehat{\uptheta_i}\right)}^2$ where θ represents the true parameter value (in the simulated data), and $\widehat{\theta_i}$ represents the estimated value of the parameter for the i^th^ bootstrap realization.

When a parameter can be estimated with low MSE and narrow confidence, this suggests that the parameter is identifiable from the model. On the other hand, larger confidence intervals or larger MSE values may be suggestive of non-identifiability.

## Results

### Model 1: Simple SEIR

Additional files 1, 2 and 3: illustrate the empirical distributions of the estimated parameters, where Additional file 1: represents the results for $\widehat{\varTheta}$_1_(β only), Additional file 2**:** for $\widehat{\varTheta}$_2_ (β and γ), and Additional file 3**:** for $\widehat{\varTheta}$_3_ (β, γ, and κ). The figures also show the original simulated data and the 200 simulated datasets for each estimated parameter set.

**Fig. 5:**
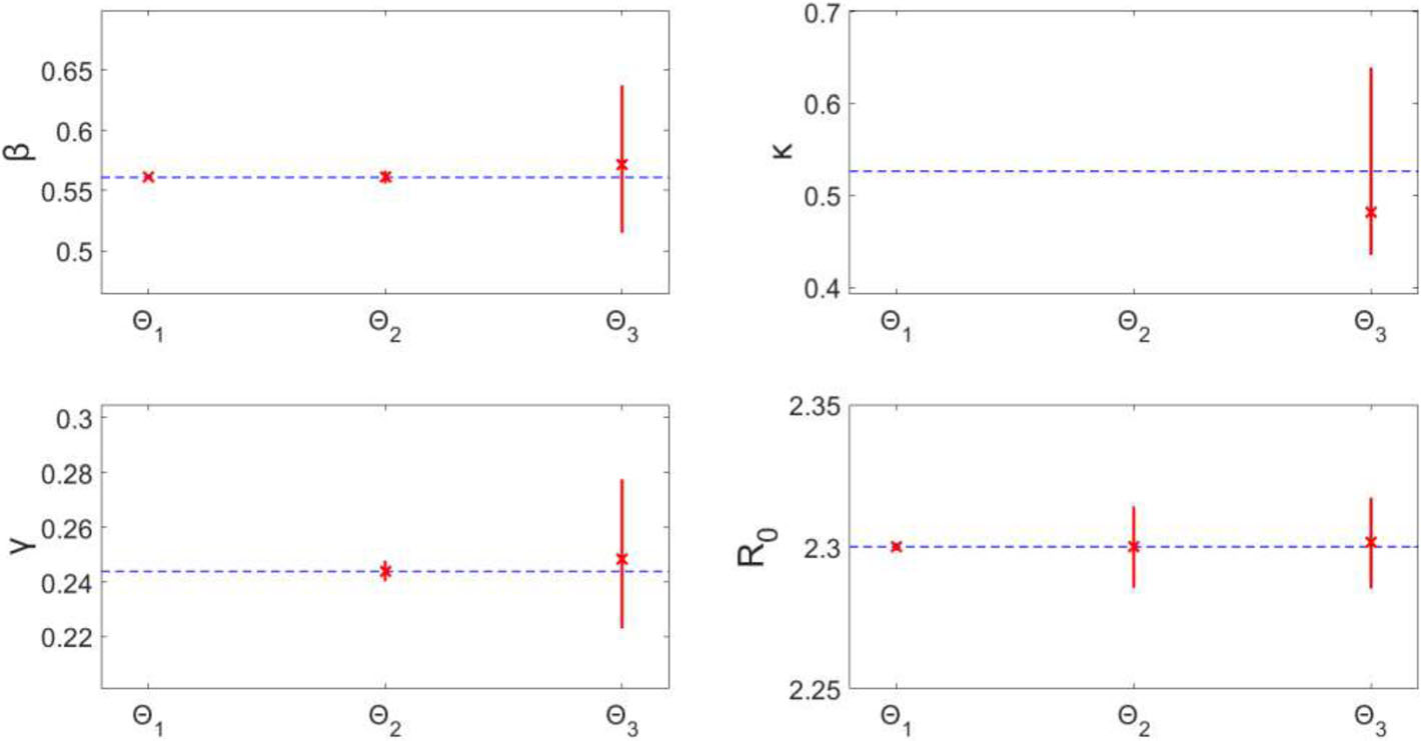
Model 1–95% confidence intervals (vertical red lines) for the distributions of each estimated parameter obtained from the 200 realizations of the simulated datasets. Mean estimated parameter value is denoted by a red x, and the true parameter value is represented by the blue dashed horizontal line. Θ_i_ denotes the estimated parameter set, where i indicates the number of parameters being jointly estimated

**Fig. 6:**
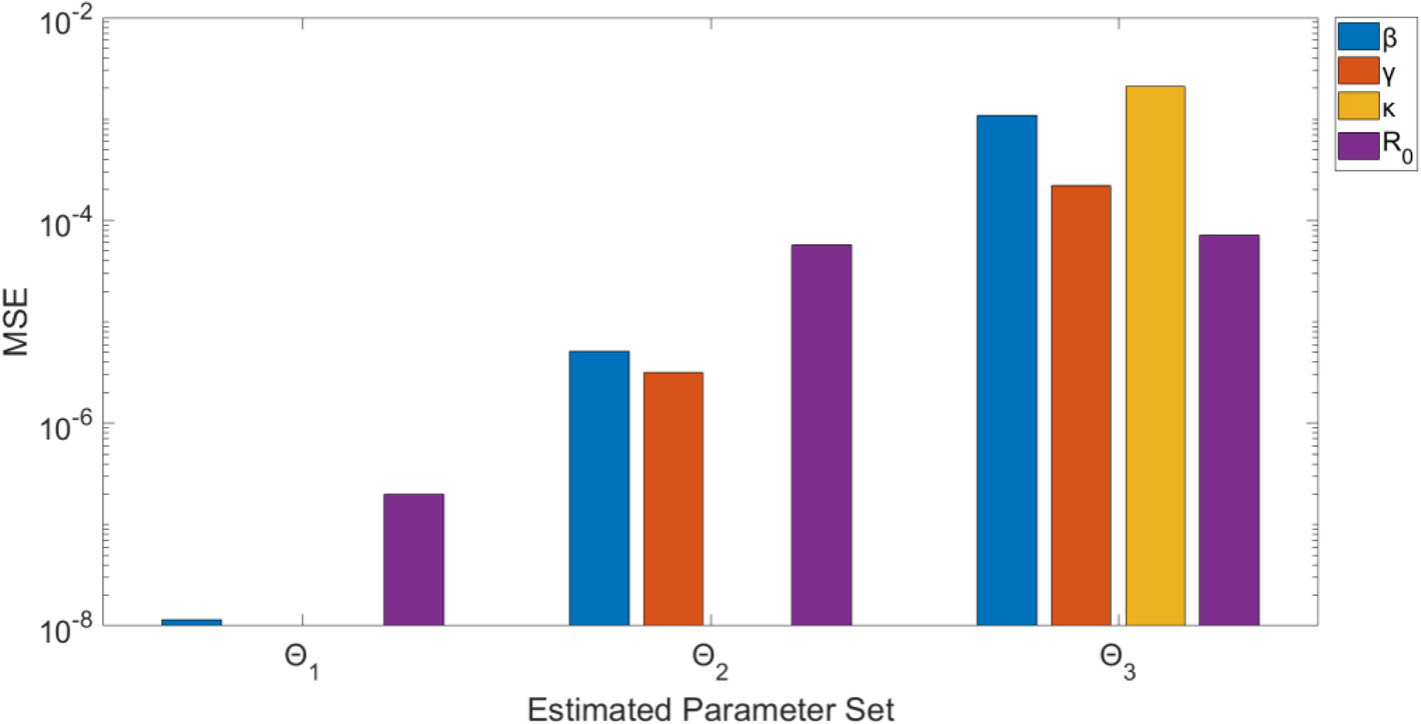
Model 1 – Mean squared error (MSE) of the distribution of parameter estimates (200 realizations) for each estimated parameter set Θ_i_, where i indicates the number of parameters being jointly estimated. Note that the y-axis (MSE) is represented with a logarithmic scale

Estimating only β (Θ_1_), results in precise (small confidence interval range) and unbiased (small MSE) estimates of β. Similarly, estimating β and γ (Θ_2_) provides precise and unbiased estimates for both parameters. The precision of the estimates can be seen in Fig. 5: the confidence intervals for the estimates (represented by red vertical lines) remain close to the true parameter value (blue horizontal dotted line). The MSE plot (Fig. 6) shows an MSE value of < 10^− 7^ for β in Θ_1_ and values of < 10^− 4^ for both β and γ in Θ_2_.

Simultaneously estimating all 3 parameters, β, κ, and γ (Θ_3_), results in wider confidence intervals and larger MSE than the two previous subsets. The confidence intervals for β (0.516, 0.636) and γ (0.223, 0.277) have a narrow range and enclose the true values of the parameters. The MSE for these two are larger compared to the previous subsets, though all MSE values are < 10^− 2^. The confidence interval for κ has a slightly larger range (0.440, 0.613), though this correlates with a small latent period difference of less than a day. Also, the MSE for κ is comparable to the other parameters. This indicates that all three parameters can be identified from daily incidence data of the epidemic curve with Poisson error structure.

Moreover, R_0_ can be estimated precisely with unbiased results. Despite the larger confidence intervals for the other parameters estimated in Θ_3_ (compared to Θ_1_, Θ_2_), the range around R_0_ is still very precise: (2.286, 2.317). Similarly, MSE for R_0_ is < 10^− 4^ for all runs. This indicates that the estimates of R_0_ are robust to variation or bias in the other parameter estimates – we will continue to explore this theme in the proceeding models.

### Model 2: SEIR with asymptomatic and hospitalized/diagnosed and reported

**Fig. 7:**
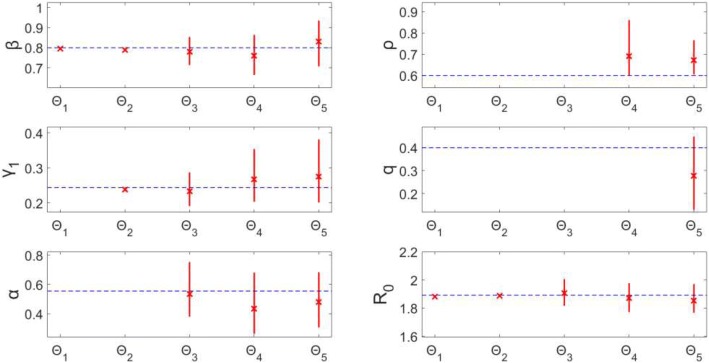
Model 2–95% confidence intervals (vertical red lines) for the parameter estimate distributions obtained from the 200 realizations of the simulated datasets. Mean estimated parameter value is denoted by red x, and the true parameter value is represented by the blue dashed horizontal line. Θ_i_ denotes the estimated parameter set, where i indicates the number of parameters being jointly estimated

**Fig. 8:**
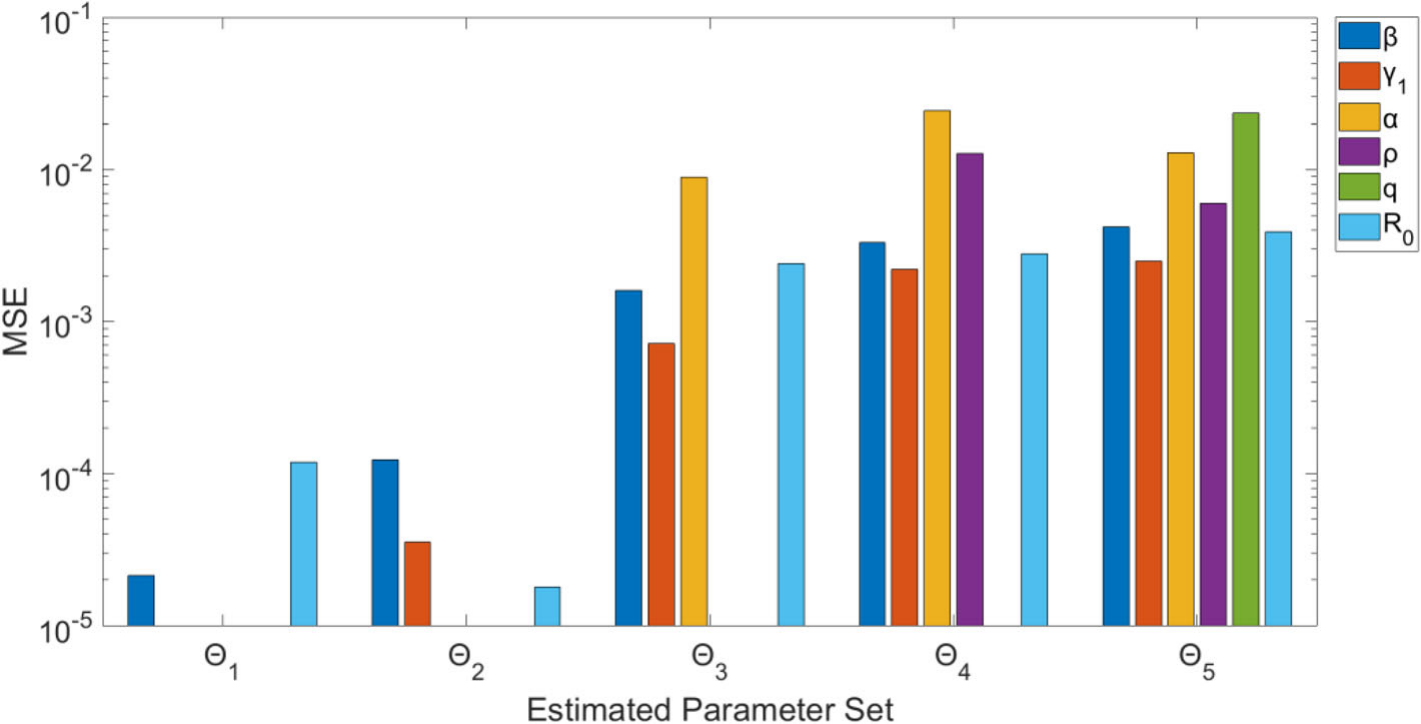
Model 2 – Mean squared error (MSE) of the distribution of parameter estimates (200 realizations) for each estimated parameter set Θ_i_, where i indicates the number of parameters being jointly estimated. Note that the y-axis (MSE) is represented with a logarithmic scale

Estimating β only (Θ_1_) or β and γ_1_ (Θ_2_) provides precise estimates with small MSE (Figs. 7 & 8). For each Θ_i_ (where *i* > 2), each additional parameter being estimated corresponds with, on average, a larger confidence interval range and higher MSE for each estimated parameter. Essentially, for each parameter, the uncertainty grows with the number of other parameters being jointly estimated. Θ_3_, estimating β, γ_1_, and α, provides estimates of β and γ_1_ with relatively small confidence ranges (95% CI: (0.717, 0.851), (0.192, 0.286), respectively) and MSE values (MSE = 0.0016, 7.15*10^− 4^, respectively); however, estimates for α produce a wider range of values (0.386, 0.748), as well as an MSE value over 5 times higher than the other parameters (MSE = 0.0089), though still < 10^− 2^.

Results for Θ_4_ and Θ_5_ indicate that none of the parameters can be well-identified from case incidence data while simultaneously estimating > 3 parameters. For each, multiple parameters have MSE values > 10^− 2^ (Fig. 8), and the confidence intervals are comparatively wide. Additionally, the confidence intervals for ρ (Θ_4_: (0.602, 0.858); Θ_5_: (0.608, 0.763)) do not include the true value of 0.60.

Looking at confidence intervals and MSE (Figs. 7 & 8) for R_0_, we find again that R_0_ is identifiable across each Θ_i_. The confidence intervals for R_0_ all have a range < 0.2, and the MSE values for each Θ_i_ are < 10^− 2^. These R_0_results are consistent with those in Model 1, despite the identifiability issues of other parameters seen here in Model 2. This is an important result, indicating that even when identifiability issues exist in other model parameters, we can still provide reliable estimates of R_0_ without having to know the true values of the other parameters. It also shows that while noise in the data may affect parameter estimation for some parameters, composite parameters, like R_0_, can still be accurately calculated from the same data.

### Model 3: The Legrand model (Ebola)

**Fig. 9:**
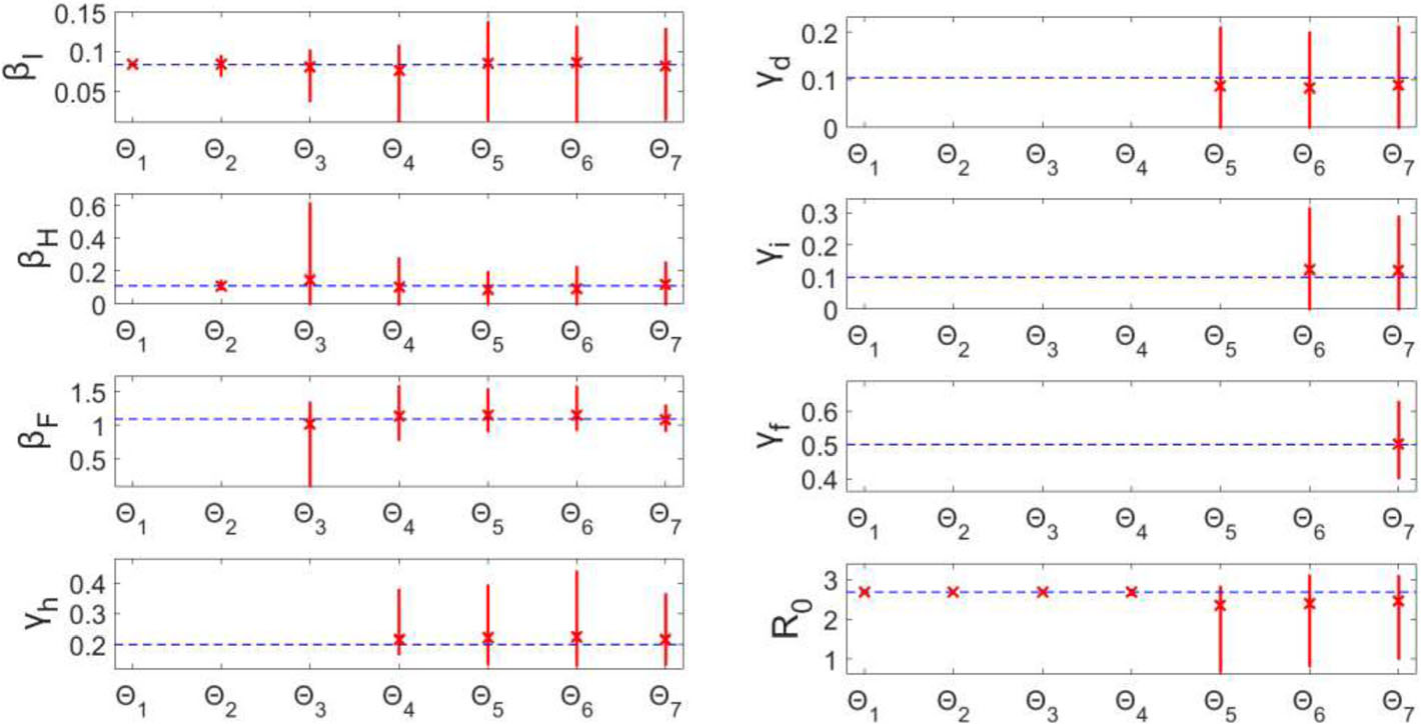
Model 3–95% confidence intervals (vertical red lines) for the parameter estimate distributions obtained from the 200 realizations of the simulated datasets. Mean estimated parameter value is denoted by red x, and the true parameter value is represented by the blue horizontal line. Θ_i_ denotes the estimated parameter set, where i indicates the number of parameters being jointly estimated

**Fig. 10:**
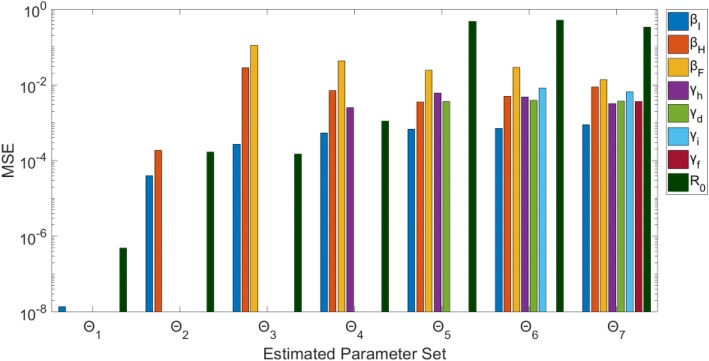
Model 3 – Mean squared error (MSE) of the distribution of parameter estimates (200 realizations) for each estimated parameter set Θ_i_, where i indicates the number of parameters being jointly estimated. Note that the y-axis (MSE) is represented with a logarithmic scale

Estimated parameter sets Θ_1_ and Θ_2_ (β_I_ only, β_I_ and β_H_ respectively) result in unbiased (MSE < 10^− 3^), precise estimates of the parameters (Figs. 9 & 10). However, when jointly estimating all three β values (Θ_3_), only β_I_ is identifiable – the confidence interval is a finite range: (0.038, 0.102) and the estimates are unbiased (MSE = 2.71*10^− 4^). Parameters β_H_ (0, 0.614) and β_F_ (0.097, 1.341) both have wide confidence intervals indicating uncertainty suggestive of non-identifiability. Estimating four parameters (Θ_4_), only β_H_ is identifiable with a small range and bias; whereas, the remaining three parameter estimates have larger confidence intervals (Fig. 9).

For Θ_i_ where *i* > 4, none of the parameters can be identified from the model/data. Each parameter (for runs Θ_5_ – Θ_7_) has either a large confidence range and/or comparatively large MSE. Some parameters have MSE values < 10^− 2^ (Fig. 10), but the wide range of uncertainty around these parameters is still indicative of non-identifiability (Fig. 9).

Remarkably, R_0_ can be precisely estimated with unbiased results for parameter sets Θ_1_ – Θ_4_ (Figs. 9 & 10). When simultaneously estimating five or more parameters, however, the associated uncertainty of all the parameters results in non-identifiability of R_0_. For Θ_5_, for example, R_0_ estimates vary widely in the range (0.683, 2.821) with an MSE of 0.467. As previously mentioned, R_0_ is a threshold parameter (epidemic threshold at R_0_ = 1), so given the confidence interval including the critical value 1, we would not have the ability to distinguish between the potential for epidemic spread versus no outbreak.

### Model 4: Zika model with human and mosquito populations

**Fig. 11:**
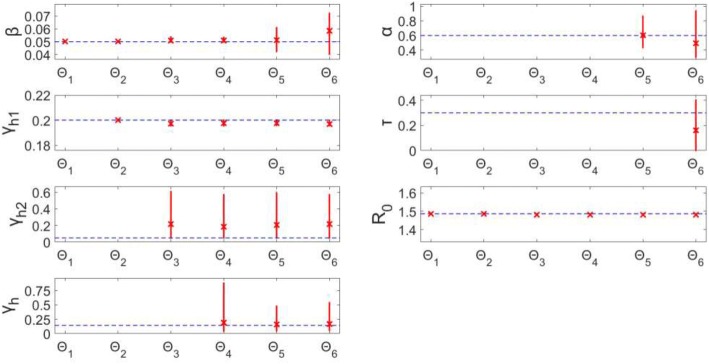
Model 4–95% confidence intervals (vertical red lines) for the parameter estimate distributions obtained from the 200 realizations of the simulated datasets. Mean estimated parameter value is denoted by red x, and the true parameter value is represented by the blue horizontal line. Θ_i_ denotes the estimated parameter set, where i indicates the number of parameters being jointly estimated

**Fig. 12:**
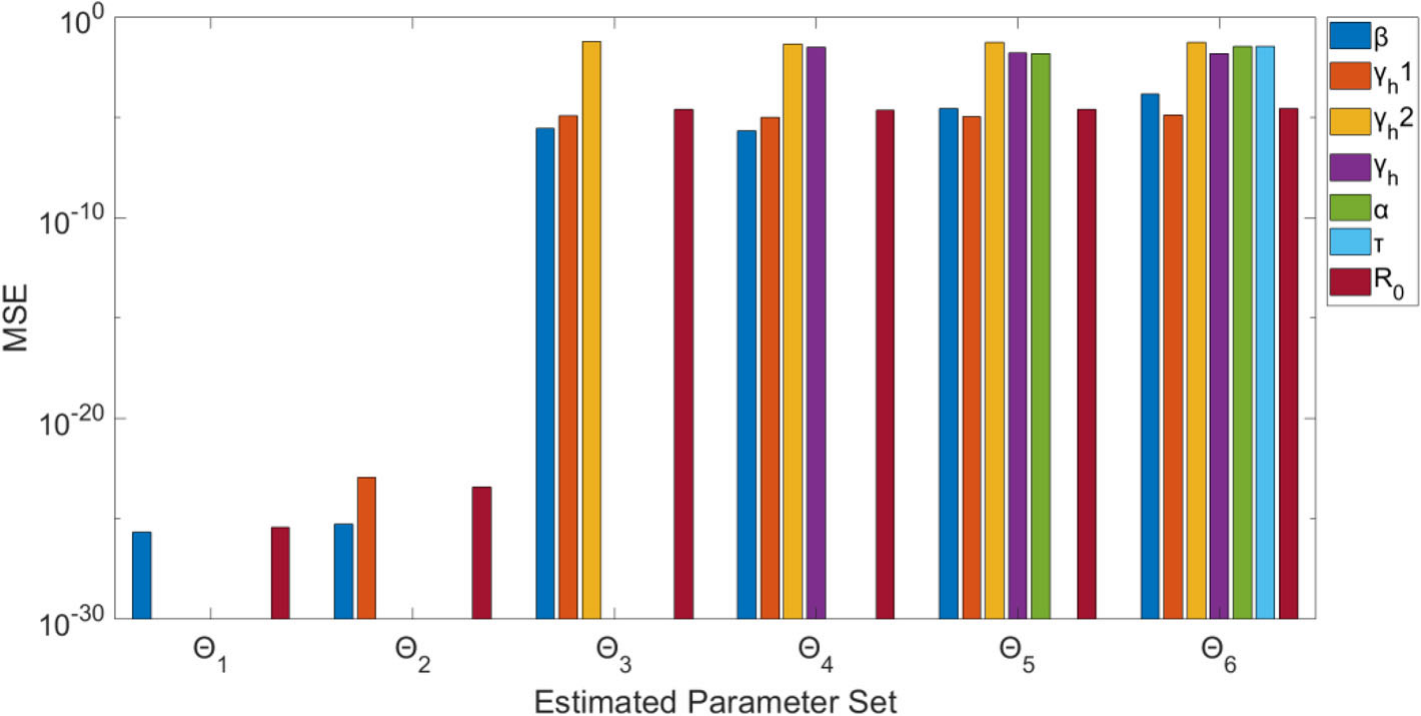
Model 4 – Mean squared error (MSE) of the distribution of parameter estimates (200 realizations) for each estimated parameter set Θ_i_, where i indicates the number of parameters being jointly estimated. Note that the y-axis (MSE) is represented with a logarithmic scale

For this complex model, we find again that when estimating only 1 or 2 parameters (Θ_1_, Θ_2_), the parameters can be recovered precisely with unbiased results (Figs. 11 & 12). When jointly estimating more than two parameters (Θ_i_: *i* > 2), non-identifiability issues arise. It can be seen that the confidence intervals and MSE for β and γ_h1_ are very small, and thus they are identifiable. However, all of the confidence intervals and MSE values for each of the other parameters (Θ_i_: i > 2) are representative of non-identifiability. The parameter estimates have a large amount of uncertainty, represented by the large confidence intervals, and are also biased estimates of the true value: MSE > 10^− 2^ for all.

In terms of R_0_, we can see that this composite parameter of interest is identifiable for all Θ_i_ (Figs. 11 & 12). Despite the large confidence intervals associated with some parameters (ex: Θ_6_ – γ_h2_: (0.047, 0.573)), when estimating more than two parameters, R_0_ can still be estimated with low uncertainty: (Θ_6_ – R_0_: (1.480, 1.486)). The R_0_ estimates have little error, as MSE < 10^− 4^ for all Θ_i_. This is consistent with the previous models in that R_0_ estimates are robust to the uncertainty and bias of the other estimated parameters.

## Discussion

In this paper we have introduced a simple computational approach for assessing parameter identifiability in compartmental models comprised of systems of ordinary differential equations. We have demonstrated this approach through various examples of compartmental models of infectious disease transmission and control. Using simulated time series of the number of new infectious individuals, we analyzed the identifiability of model characterizing transmission and the natural history of the disease. This type of analysis based on simulated data provides a crucial step in infectious disease modeling, as inferences based on estimates of non-identifiable parameters can lead to incorrect or ineffective public health decisions. Parameter identifiability and uncertainty analyses are essential for assessing the stability of the parameter estimates. Hence, it is important for researchers to be mindful that a good fit to the data does not imply that parameter estimates can be reliably used to evaluate hypotheses regarding transmission mechanisms. Moreover, quantifying the uncertainty surrounding parameter estimates is key when making inferences that guide public health policies or interventions.

Our bootstrap-based approach is sufficiently general to assess identifiability for compartmental modeling applications. We have shown that this method works well for models of varying levels of complexity, ranging from a simple SEIR model with only a few parameters (Model 1) to a complex, dual-population compartmental model with a total of 16 parameters (Model 4). Other methods exist to conduct parameter identifiability analyses. Some methods, such as Taylor series methods [15, 16] and differential algebra-based methods [17, 18], require more mathematical analyses, which becomes increasingly complicated as model complexity increases. Other methods rely on constructing the profile likelihood for each of the estimated parameters to assess local structural identifiability [11, 14, 31, 32]. In this method, one of the parameters (θ_i_) is fixed across a range of realistic values, and the other parameters are refit to the data using the likelihood function of θ_i_. Thus, identifiability of the parameters is determined by the shape of the resulting likelihood profile. Depending on the assumptions of the error structure in the data and as models become increasingly more complex, derivation of the likelihood profile and confidence intervals becomes increasingly more difficult.

Overall, our analyses indicate that parameter identifiability issues are more likely to arise with more complex models (based on number of equations/states and parameters). For example, a set of 3 parameters (Θ_3_) can be estimated with low uncertainty and bias from a simple model, like Model 1; however, for more complex models (Model 3, Model 4), estimating only 3 parameters from a single curve of case incidence resulted in lack of identifiability for at least one of the parameters in the set (Θ_3_). Also, for Θ_i_ (recall: i represents number of parameters being jointly estimated), as *i* increases, the uncertainty surrounding estimated parameters tended to increase, on average, as well (Fig. 7). One strategy to resolve parameter identifiability issues consists of restricting the number of parameters being jointly estimated while fixing other parameter values and conducting sensitivity analyses.

Importantly, we found that R_0_ is a robust composite parameter, even in the presence of identifiability issues affecting individual parameters in the model. In Model 4, despite large confidence intervals and larger MSE for the estimated parameters, R_0_ estimates were contained in a finite confidence interval with little bias (Figs. 11 & 12). For example, for parameter set Θ_6_, only two of the estimated parameters could be reliably identified from the data, yet R_0_ could be identified with little uncertainty or bias. These findings are in line with the identifiability results of R_0_ for a vector-borne disease model (similar to Model 4), even when other model parameters could not be properly estimated [14]. R_0_ is often a parameter of interest, as R_0_ values have been related to the size or impact of an epidemic [1]. Moreover, R_0_ estimates can be used to characterize initial transmission potential, assess the risk of an outbreak, and evaluate the impact of potential interventions, so it is beneficial to know we can reliably obtain R_0_ estimates, despite lack of identifiability in other parameters.

It is important to emphasize that our methodology is helpful to uncover identifiability issues which could arise from 1) the lack of information in the data or 2) the structure of the model. We also note that our examples assess identifiability of parameters by relying on the entire curve of incidence data of a single epidemic. Future work could include identifiability analyses in the context of limited data using different sections of the trajectory of the outbreak. We also assume that only one model variable (state) is observed, so future analyses could incorporate more than one observed variable to potentially improve the identifiability of parameters without changing the model. For example, for Model 3 (Ebola), the incidence curves of new hospitalized cases and new deaths could provide additional information that better constrain parameter estimates, thereby improving parameter identifiability results.

## Conclusions

For modeling studies, we recommend conducting comprehensive parameter identifiability analyses based on simulated data prior to attempting to fit the model to data. It is important to emphasize that lack of identifiability could be due to lack of information in the data or the structure of the model. The analyses also help guide the set of parameters in the model that can be jointly estimated – identifiability issues may not arise until any given number of parameters are being simultaneously estimated. If the analysis indicates non-identifiability of certain parameters, may have to be assessed in sensitivity analyses (rather than estimated) to address the identifiability issue.

In summary, the ability to make sound public health decisions regarding an infectious disease outbreak is crucial for the general health and safety of a population. Knowledge of whether a parameter is identifiable from a given model and data is invaluable, as estimates of non-identifiable parameters should not be used to inform public health decisions. Further, parameter estimates should be presented with quantified uncertainty. The methodology presented in this paper adds to the essential toolkit for conducting model-based inferences.

## Additional files

Additional file 1: Model 1 – Θ_1_ (estimating β only): The histograms display the empirical distributions of the parameter estimates using 200 bootstrap realizations, where the solid red horizontal line represents the 95% confidence interval for parameter estimates, and the dashed red vertical line indicates the true parameter value. Note, κ and γ are set to their true values in the data. The bottom left graph shows the data from the model (blue circles), and 200 realizations of the epidemic curve assuming a Poisson error structure (light blue lines). The solid red line corresponds to the best-fit of the model to the data, and the dashed red lines correspond to the 95% confidence bands around the best fit. (TIF 5423 kb)

Additional file 2: Model 1 – Θ_2_ (estimating β and γ): The histograms display the empirical distributions of the parameter estimates using 200 bootstrap realizations, where the solid red horizontal line represents the 95% confidence interval for parameter estimates, and the dashed red vertical line indicates the true parameter value. Note, κ is set to the true value from the data. The bottom left graph shows the data from the model (blue circles), and 200 realizations of the epidemic curve assuming a Poisson error structure (light blue lines). The solid red line corresponds to the best-fit of the model to the data, and the dashed red lines correspond to the 95% confidence bands around the best fit. (TIF 5423 kb)

Additional file 3: Model 1 – Θ_3_ (estimating β, κ, and γ): The histograms display the empirical distributions of the parameter estimates using 200 bootstrap realizations, where the solid red horizontal line represents the 95% confidence interval for parameter estimates, and the dashed red vertical line indicates the true parameter value. The bottom left graph shows the data from the model (blue circles), and 200 realizations of the epidemic curve assuming a Poisson error structure (light blue lines). The solid red line corresponds to the best-fit of the model to the data, and the dashed red lines correspond to the 95% confidence bands around the best fit. (TIF 5423 kb)
