# Supplementary figures and images for: Assessing parameter identifiability in compartmental dynamic models using a computational approach: application to infectious disease transmission models
Source: Theor Biol Med Model. 2019 Jan 14;16:1. doi: 10.1186/s12976-018-0097-6 (PMC6332839; doi:10.1186/s12976-018-0097-6)

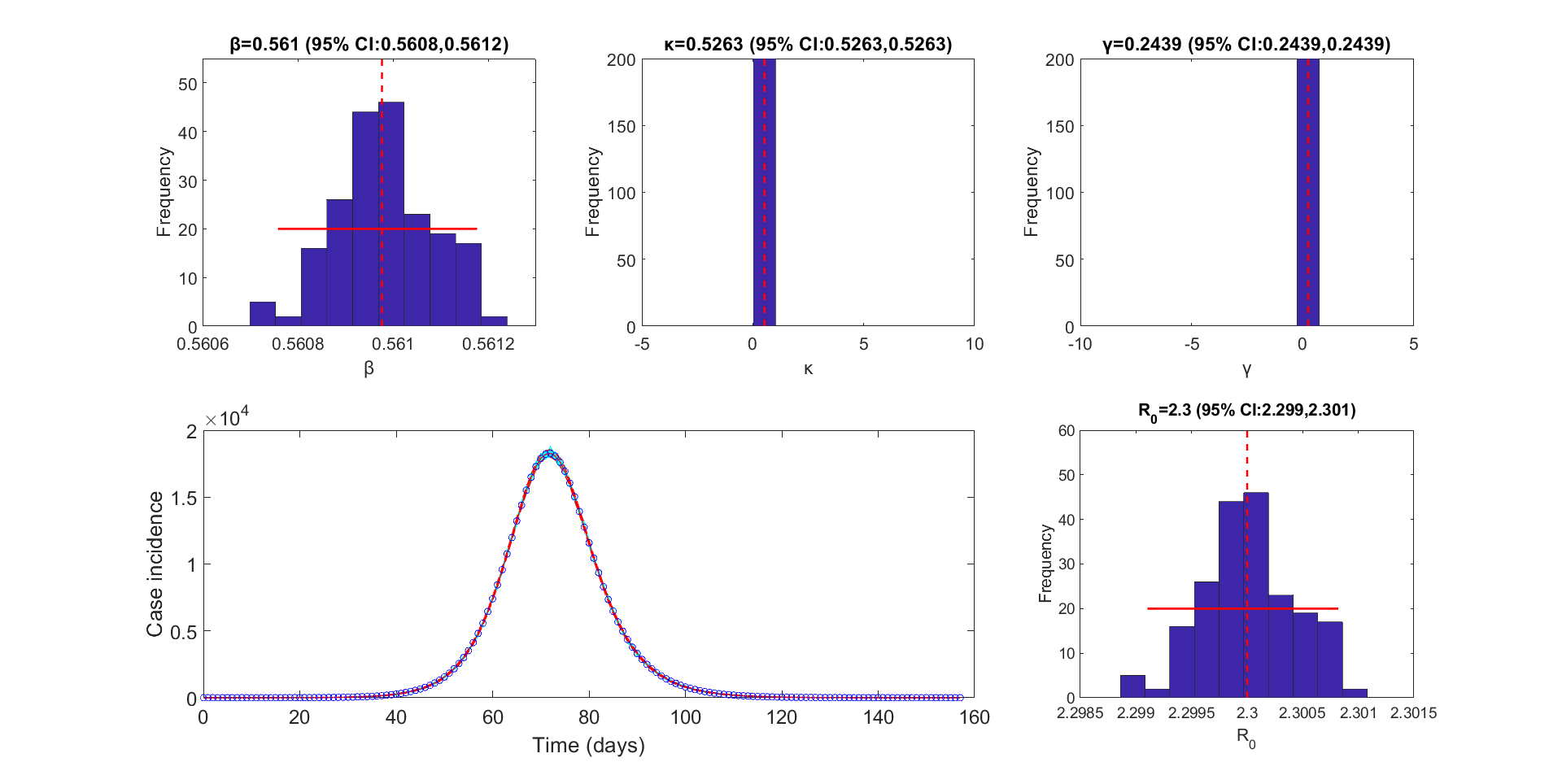

Supplement: Supplementary file 1 — Model 1 – Θ1 (estimating β only): The histograms display the empirical distributions of the parameter estimates using 200 bootstrap realizations, where the solid red horizontal line represents the 95% confidence interval for parameter estimates, and the dashed red vertical line indicates the true parameter value. Note, κ and γ are set to their true values in the data. The bottom left graph shows the data from the model (blue circles), and 200 realizations of the epidemic curve assuming a Poisson error structure (light blue lines). The solid red line corresponds to the best-fit of the model to the data, and the dashed red lines correspond to the 95% confidence bands around the best fit. (TIF 5423 kb) [file 12976_2018_97_MOESM1_ESM.tif]

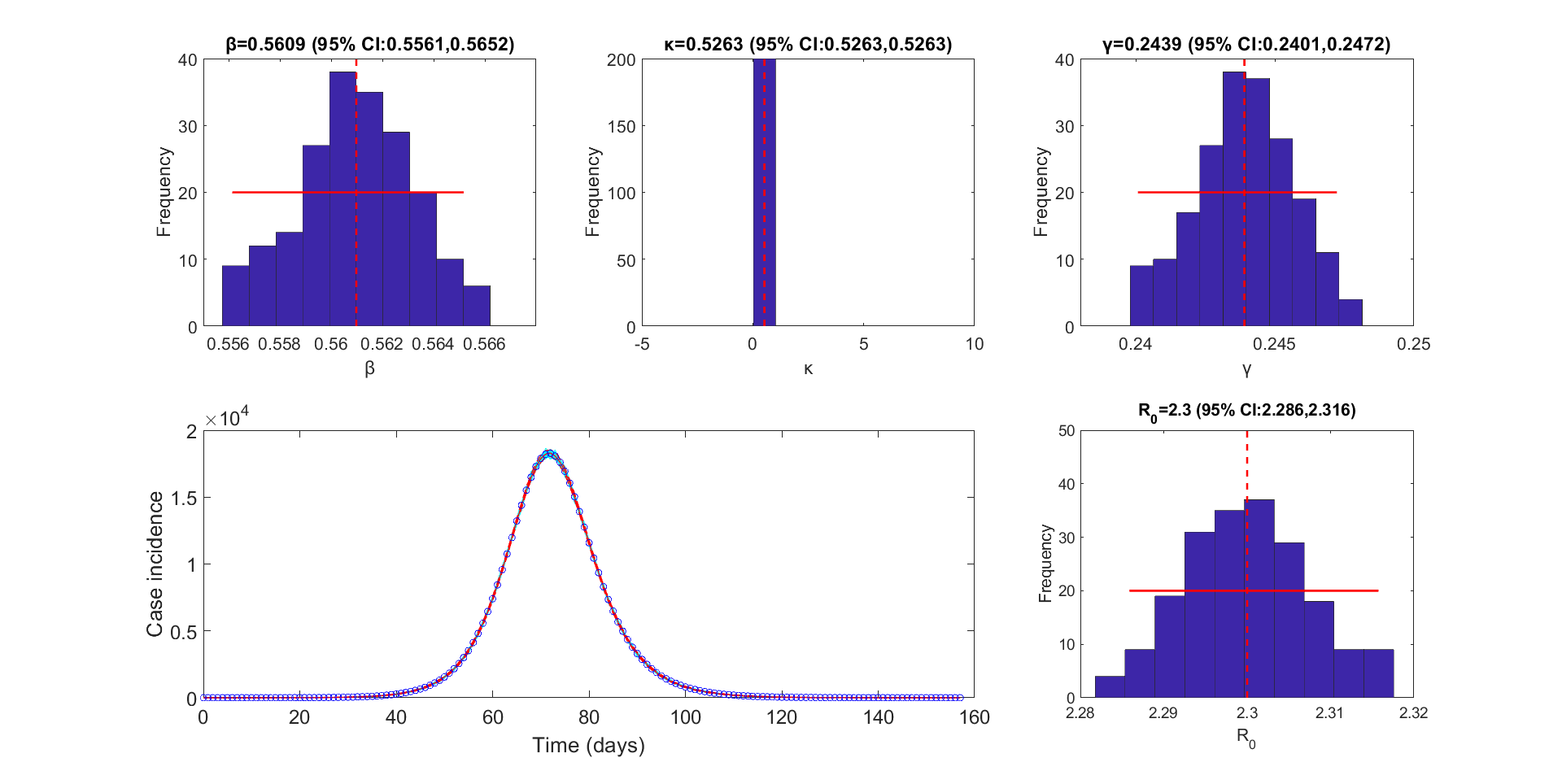

Supplement: Supplementary file 2 — Model 1 – Θ2 (estimating β and γ): The histograms display the empirical distributions of the parameter estimates using 200 bootstrap realizations, where the solid red horizontal line represents the 95% confidence interval for parameter estimates, and the dashed red vertical line indicates the true parameter value. Note, κ is set to the true value from the data. The bottom left graph shows the data from the model (blue circles), and 200 realizations of the epidemic curve assuming a Poisson error structure (light blue lines). The solid red line corresponds to the best-fit of the model to the data, and the dashed red lines correspond to the 95% confidence bands around the best fit. (TIF 5423 kb) [file 12976_2018_97_MOESM2_ESM.tif]

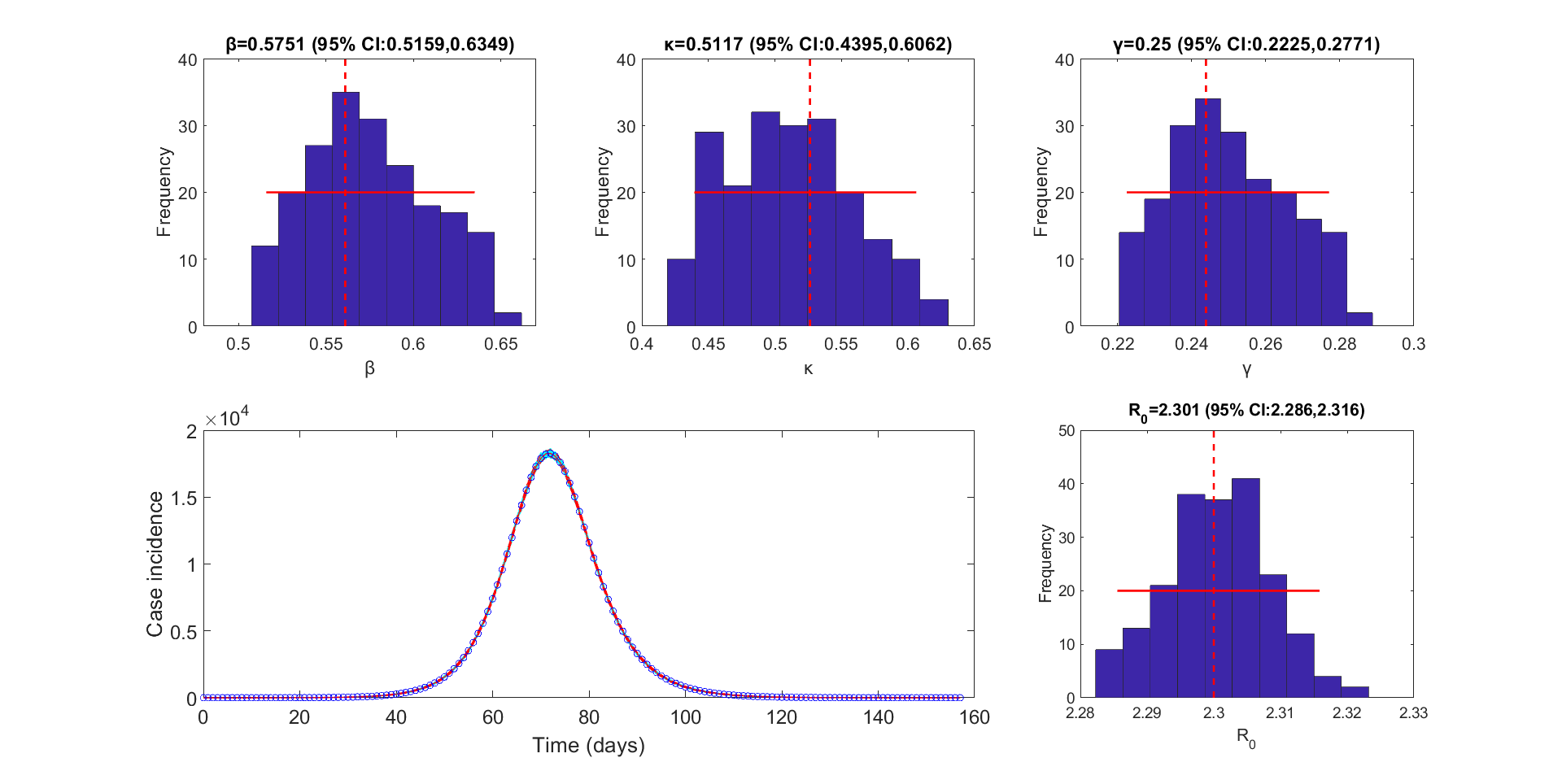

Supplement: Supplementary file 3 — Model 1 – Θ3 (estimating β, κ, and γ): The histograms display the empirical distributions of the parameter estimates using 200 bootstrap realizations, where the solid red horizontal line represents the 95% confidence interval for parameter estimates, and the dashed red vertical line indicates the true parameter value. The bottom left graph shows the data from the model (blue circles), and 200 realizations of the epidemic curve assuming a Poisson error structure (light blue lines). The solid red line corresponds to the best-fit of the model to the data, and the dashed red lines correspond to the 95% confidence bands around the best fit. (TIF 5423 kb) [file 12976_2018_97_MOESM3_ESM.tif]
